# Ιdiosyncratic Non-Cardiogenic Pulmonary Edema Following Acetazolamide Administration: A Case Report and Review of Pathogenic Mechanisms

**DOI:** 10.3390/reports9020107

**Published:** 2026-03-30

**Authors:** Athanasia-Marina Peristeri, Fotini Ampatzidou, Ioanna-Maria Mouskeftara, Olympia Akritidou, Anastasios Tsangaleas, Christina Chrysanthi Theocharidou, Athina Lavrentieva

**Affiliations:** 1 Department of Internal Medicine, George Papanikolaou General Hospital of Thessaloniki, 57010 Thessaloniki, Greece; ak.olympia@gmail.com; 2 1st Intensive Care Unit, George Papanikolaou General Hospital of Thessaloniki, 57010 Thessaloniki, Greece; fampatzidou@gmail.com (F.A.); sesillianna@hotmail.com (I.-M.M.); anastasiostsangaleas@yahoo.com (A.T.); chrtheoch@hotmail.gr (C.C.T.); alavrenti@gmail.com (A.L.)

**Keywords:** acetazolamide, non-cardiogenic pulmonary edema (NCPE), endothelial injury, intraocular pressure (IOP)

## Abstract

**Background and Clinical Significance:** Acetazolamide is routinely used post-cataract surgery to prevent intraocular pressure (IOP) spikes. Rare non-cardiogenic pulmonary edema (NCPE) cases highlight its risks in elderly comorbid patients. This report details acetazolamide-induced NCPE and provides a review of current evidence from the literature. **Case Presentation:** A 74-year-old male with chronic kidney disease, atrial fibrillation, and aortic aneurysm repair received 250 mg oral acetazolamide post-cataract extraction. Clinical, imaging, and lab data were documented during Intensive Care Unit (ICU) stay. PubMed/Google Scholar review identified similar cases. Within 30 min, severe hypoxemia with SpO_2_ (peripheral oxygen saturation) of 77%, accompanied by tachypnea and hypertension, necessitated endotracheal intubation. Echocardiography showed preserved left ventricular (LV) function; computed tomography (CT) confirmed bilateral alveolar opacities without cardiomegaly or embolism, indicating permeability-mediated NCPE. Lung-protective mechanical ventilation and vasopressor therapy resulted in hemodynamic and respiratory stabilization. On day 4, ventilator-associated pneumonia (VAP) due to *Acinetobacter baumannii* resolved with targeted antibiotic therapy. The patient made a full recovery following ICU discharge. To date, nine prior cases have been reported, alongside 31 entries in EudraVigilance reflecting a 19.4% mortality rate. **Conclusions:** Rapid-onset NCPE from acetazolamide involves endothelial injury, distinct from cardiogenic pulmonary edema. Early recognition, drug cessation, and admission to the intensive care unit (ICU) are vital components of therapeutic intervention. Risk stratification and pharmacovigilance are recommended for perioperative safety.

## 1. Introduction and Clinical Significance

Acetazolamide is a sulfonamide-derived carbonic anhydrase inhibitor widely used for the prevention and management of IOP elevation following cataract or glaucoma surgery [1]. Its mechanism of action involves reversible inhibition of carbonic anhydrase in multiple tissues, including the ocular epithelium. In the ciliary body of the eye, inhibition of carbonic anhydrase decreases bicarbonate-dependent aqueous humor production, thus lowering intraocular pressure. As such, it is considered standard prophylaxis in many cataract perioperative protocols, especially in elderly patients with risk factors for IOP elevation (e.g., pseudoexfoliation, narrow angles) [1].

Despite being generally well tolerated, acetazolamide has been associated with numerous side effects, the majority of which are mild and include paresthesia, fatigue, dysgeusia, or electrolyte imbalance. Rarely, however, serious systemic reactions may occur, including Stevens–Johnson syndrome, hepatic necrosis, and NCPE may occur [2]. Published reports document that such adverse reactions typically emerge within a relatively short period, ranging from a few minutes up to one hour after ingestion of the standard prophylactic dose, which is routinely administered to manage postoperative IOP following cataract surgery [2,3].

The pathogenesis of acetazolamide-induced NCPE is not yet completely understood. A widely accepted hypothesis involves three proposed mechanisms: immune-mediated capillary endothelial injury causing increased vascular permeability and alveolar flooding [4,5]; idiosyncratic endothelial damage from sulfonamide cross-reactivity [6]; and a capillary-leak phenomenon similar to drug-induced acute respiratory distress syndrome (ARDS) with endothelial dysfunction and alveolar-capillary barrier disruption [7]. Distinctly, this condition develops without LV systolic dysfunction, elevated pulmonary capillary wedge pressure, or fluid overload, differentiating it from cardiogenic pulmonary edema [8]. Patients typically present with rapid dyspnea, oxygen desaturation, and bilateral alveolar infiltrates following drug exposure, reflecting immune-mediated and endothelial mechanisms rather than hydrostatic pressure overload [4].

The following report presents a case of a 74-year-old male who developed acute, severe NCPE within one hour of receiving a single 250 mg oral dose of acetazolamide following uncomplicated cataract surgery. Given the patient’s clinical history, critical care management, and diagnostic workup, it is crucial to identify this uncommon but potentially lethal reaction in older patients having eye surgery.

## 2. Case Presentation

A 74-year-old Caucasian male with a medical history of chronic kidney disease, paroxysmal atrial fibrillation, hypothyroidism, and previous surgical repair of an ascending aortic aneurysm presented with acute hypoxemic respiratory failure requiring ICU admission within hours of an uncomplicated elective cataract extraction. Prior to surgery, the patient was asymptomatic with no respiratory complaints, and his perioperative management was limited to continuation of his baseline home medications without administration of intravenous fluids or supplemental pharmacologic agents. Following completion of the ophthalmic procedure, prophylactic acetazolamide was administered per institutional protocol for the prevention of postoperative IOP elevation.

Approximately 30 minutes following acetazolamide administration, the patient developed nausea and progressive tachypnoea. Initial clinical assessment revealed hypertension with systolic blood pressure of 179 mmHg and diastolic blood pressure of 98 mmHg, sinus rhythm at 98 beats per minute, marked respiratory distress with tachypnoea of approximately 40 breaths per minute, and SpO_2_ of 77% on ambient air. The patient was afebrile with an axillary temperature of 36.7 °C. Physical examination demonstrated diminished breath sounds bilaterally with basilar crackles and prolonged expiratory phase on chest auscultation, alongside jugular venous distension. He was subsequently endotracheally intubated in the context of rapidly progressive respiratory deterioration necessitating transition to controlled mechanical ventilation, given escalating work of breathing, refractory hypoxemia, and the imminent risk of ventilatory failure despite supplemental oxygen and initial supportive measures.

**Figure 1:**
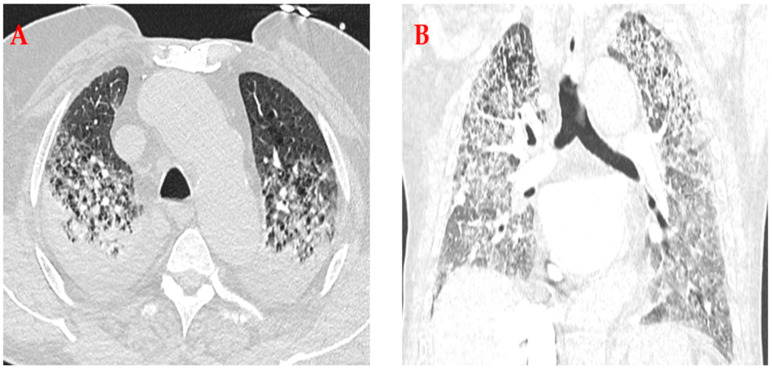
CT of the chest showing interstitial pulmonary oedema and bilateral pleural effusions (**A**) Transverse and (**B**) Coronal.

Upon admission to the ICU, he was receiving controlled mechanical ventilation with tidal volume of 6 mL/kg and Positive End-Expiratory Pressure (PEEP) of 8 mmHg, hemodynamically supported with low-dose vasopressor infusion, and sedated to facilitate ventilator synchrony. Examination showed equal chest rise, diffuse bilateral end-inspiratory crackles, and no peripheral edema; heart sounds were regular without new murmurs, and capillary refill was preserved. Initial laboratory testing on admission demonstrated a normal leukocyte count and no elevation in C-reactive protein, consistent with a non-infectious inflammatory process (Table 1). Further laboratory investigation revealed low natriuretic peptide levels, stable troponin without dynamic change, and renal indices at the patient’s chronic baseline. Arterial blood gases on controlled ventilation demonstrated a reduced PaO_2_/FiO_2_ ratio with significant impairment of oxygenation. Portable chest radiography revealed bilateral, diffuse alveolar–interstitial opacities with dependent predominance, without cardiomegaly, vascular cephalization, or Kerley B lines. Point-of-care lung ultrasound showed diffuse, confluent B-lines in multiple zones with small bilateral pleural effusions; dynamic air bronchograms were present in dependent regions, consistent with alveolar flooding rather than pure atelectasis. Focused transthoracic echocardiography demonstrated a preserved LV ejection function of 60% without new wall-motion abnormalities or significant diastolic dysfunction to account for the severity of pulmonary edema; right ventricular size/function were within normal limits aside from mild pressure load, with moderate tricuspid regurgitation, normal morphology and function of the remaining valves, and no pericardial effusion. CTPA showed no intraluminal filling defects in the main, lobar, or segmental pulmonary arteries, thereby excluding acute pulmonary embolism. Bilateral interstitial pulmonary edema with pleural effusions was identified, which in the context of preserved cardiac function, normal BNP levels, and close temporal association with acetazolamide administration, was consistent with NCPE (Figure 1).

The above findings were most consistent with permeability-mediated NCPE temporally associated with recent acetazolamide exposure. The patient was therefore managed with controlled mechanical ventilation using low tidal volumes and moderate PEEP, with careful titration of the fraction of FiO_2_ to maintain adequate oxygenation while minimizing ventilator-induced lung injury. Low-dose vasopressor support was also administered to ensure adequate tissue perfusion during the acute phase.

On the fourth day of ICU hospitalization, the patient developed fever, leucocytosis, and elevated inflammatory markers with increased oxygen requirements and purulent secretions. These findings were consistent with VAP as a nosocomial complication of ICU stay. Empirical broad-spectrum antimicrobial therapy with piperacillin–tazobactam and linezolid was initiated pending microbiological results and antimicrobial susceptibility testing. Microbiological evaluation yielded multidrug-resistant *Acinetobacter baumannii*, initially from bronchial samples and subsequently from blood cultures, establishing VAP with secondary bacteraemia. In consultation with infectious diseases, antimicrobial therapy was centered on high-dose ampicillin–sulbactam and tigecycline, with adjunctive agents considered according to in vitro susceptibility testing, with additional agents selected and titrated according to susceptibility results and clinical response.

Following escalation of targeted antimicrobial therapy and supportive critical care, the patient demonstrated progressive clinical improvement. Vasopressors were successfully titrated and discontinued, permitting hemodynamic stabilization. Sedation was systematically reduced, facilitating neurological awakening and weaning from mechanical ventilation. By the time of ICU discharge, the patient achieved full consciousness with a Glasgow Coma Scale score of 15, maintained hemodynamic stability on ambient supplementation with nasal oxygen at 4–5 L/min, and transitioned to partial oral intake with complementary nasogastric tube feeding. Daily physiotherapy and mobilization contributed to functional recovery.

He was subsequently transferred to a respiratory step-down unit, where his clinical status continued to improve, and he was ultimately discharged home in stable condition with supplemental oxygen, which was gradually tapered as tolerated.

## 3. Discussion

As mentioned above, acetazolamide is widely used in ophthalmology for prevention and management of IOP elevation following cataract or glaucoma surgery [1]. It is considered standard prophylaxis in perioperative cataract protocols, particularly in elderly patients at risk for postoperative pressure spikes, and is generally well tolerated with a favorable safety profile [9]. Despite its widespread use and overall safety, rare but serious systemic adverse reactions have been documented, including but not limited to acute respiratory failure with NCPE [3]. The pathophysiology underlying acetazolamide-induced pulmonary edema remains incompletely understood, with current understanding derived primarily from case-by-case analysis. Importantly, this adverse reaction typically manifests within minutes to one hour after ingestion of a standard 250 mg prophylactic dose, presenting with abrupt onset dyspnoea, hypoxemia, and bilateral alveolar infiltrates on chest imaging, distinguishing it from cardiogenic pulmonary edema through the absence of LV dysfunction, elevated pulmonary capillary wedge pressure, or fluid overload [4,5].

A systematic review of the medical literature was conducted using PubMed and Google Scholar to identify published reports of acute respiratory failure temporally associated with acetazolamide administration, primarily in the ophthalmic setting. The search yielded nine articles describing this complication (Table 2) [4,5,6,7,10,11,12,13,14]. Among these, three cases attributed acute respiratory failure to an allergic reaction mechanism [6,10,11]. Additionally, Gallerani et al. reported one case of acute respiratory failure attributed to a suspected allergic reaction, though NCPE could not be definitively excluded [12]. Four cases presented detailed descriptions of NCPE as the underlying mechanism and discussed potential pathophysiological pathways in their respective analyses [4,5,7,13]. Finally, one case report by Yilmaz et al. described acute respiratory failure occurring in the setting of acute cardiogenic pulmonary edema, representing a distinct etiological entity from the predominant pattern of non-cardiogenic presentations [14].

Regarding the pathogenesis of acetazolamide-induced NCPE, the most widely endorsed mechanism implicates an immune-mediated hypersensitivity reaction that provokes inflammatory injury to the pulmonary microvascular endothelium. The ensuing endothelial disruption compromises the integrity of the alveolar-capillary barrier, resulting in increased transvascular permeability and extravasation of proteinaceous plasma fluid into the alveolar spaces [5]. An alternative yet complementary hypothesis attributes the endothelial injury to an idiosyncratic reaction driven by molecular cross-reactivity between acetazolamide and other sulfonamide-containing compounds [6]. Furthermore, accumulating evidence indicates that acetazolamide may elicit a diffuse capillary-leak reaction resembling the pathophysiologic process underlying drug-associated ARDS. This phenomenon involves widespread endothelial disruption, release of proinflammatory mediators, and loss of epithelial tight junction integrity, collectively culminating in alveolar flooding and NCPE [7].

An important distinction exists between sulfonamide antibiotics and non-antibiotic sulfonamide compounds such as acetazolamide regarding the risk and mechanisms of allergic reactions. Sulfonamide antibiotics (e.g., trimethoprim-sulfamethoxazole) contain an N4-arylamine group, a chemical moiety strongly implicated in severe hypersensitivity reactions [15,16]. In contrast, non-antibiotic sulfonamide drugs including acetazolamide, lack this N4-arylamine structure and are therefore associated with substantially lower cross-reactivity risk in patients with documented sulfonamide antibiotic allergy [15,17]. However, the present case and others in the literature suggest that acetazolamide, despite its favorable safety profile in most patients, may rarely trigger severe idiosyncratic reactions through alternative pathways unrelated to classical IgE-mediated hypersensitivity, particularly in genetically or individually predisposed individuals [4,5,7,13].

The pathophysiology of acetazolamide-induced acute respiratory failure manifesting as NCPE fundamentally differs from cardiogenic edema in that it results from increased capillary permeability and endothelial injury rather than elevated pulmonary capillary wedge pressure or LV dysfunction, allowing protein-rich fluid to extravasate into the alveolar compartment [4,5,7]. The rapid temporal onset, typically within 30 min to one hour after ingestion, coupled with the absence of prior sensitization history in most cases, suggests an acute idiosyncratic rather than dose-dependent mechanism [4,5]. This permeability-mediated process results in bilateral alveolar flooding with proteinaceous edema fluid, severe hypoxemia despite preserved cardiac function, and characteristic imaging findings of bilateral infiltrates without cardiomegaly or pulmonary vascular engorgement. Therefore, it constitutes a distinct clinical and pathophysiological phenotype that demands recognition and differentiation from cardiogenic pulmonary edema in the acute setting [18,19].

Given the current evidence, acetazolamide-induced acute respiratory failure appears to fall within the broader spectrum of drug-induced pulmonary parenchymal disease (DIPPD), but with a distinct, incompletely elucidated pathophysiological profile [20]. DIPPD encompasses a heterogeneous group of entities, including hypersensitivity pneumonitis, eosinophilic pneumonias, interstitial fibrosis, diffuse alveolar hemorrhage, and NCPE. Their underlying pathophysiological mechanisms remain poorly understood and vary widely depending on the offending agent and host factors. Nevertheless, diagnosis and treatment follow common principles, such as drug withdrawal, exclusion of alternative etiologies, and selective corticosteroid therapy [21,22]. In this framework, NCPE related to acetazolamide can be conceptualized as a permeability-mediated drug-induced lung injury, in which idiosyncratic or immune-driven endothelial damage to the pulmonary microvasculature leads to abrupt transudation of protein-rich fluid into the alveolar spaces in the absence of hydrostatic overload, thereby aligning it with other forms of drug-induced parenchymal disease while preserving a distinct clinical and temporal signature that complicates recognition in everyday practice [2]. This pathophysiological distinction positions acetazolamide as a unique agent within the DIPPD spectrum, warranting separate clinical recognition and pharmacovigilance attention to optimize perioperative safety and patient outcomes [1,20].

While acetazolamide remains a widely accepted and generally safe pharmacologic option for perioperative intraocular pressure management, the present case and recent pharmacovigilance data confirm that NCPE represents a rare but potentially fatal adverse reaction that must be urgently recognized and managed. A disproportionality analysis from the EudraVigilance database identified 31 cases of acetazolamide-associated pulmonary edema, with six fatalities (19.4%) following a single 250 mg oral dose, typically within 10–90 min of administration [23]. This rapid onset and high mortality emphasize the need for clinicians, including ophthalmologists and anesthesiologists, to maintain heightened suspicion for acute dyspnea and hypoxemia following acetazolamide exposure, even without prior sensitization or hemodynamic instability. Immediate discontinuation of the drug, initiation of lung-protective ventilation, and escalation to intensive care are essential therapeutic steps to mitigate life-threatening respiratory compromise [4,5,7,13]. Given the severity of this idiosyncratic reaction, systematic pharmacovigilance reporting and preoperative risk stratification in elderly patients with comorbidities should be prioritized to prevent recurrences and improve perioperative safety outcomes.

## 4. Conclusions

In conclusion, this report highlights the critical diagnostic challenge posed by acetazolamide-induced NCPE in patients with complex comorbidity profiles. Although our patient’s history of chronic kidney disease, atrial fibrillation, and aortic aneurysm repair strongly suggested a cardiogenic etiology, a systematic workup definitively established a permeability-mediated mechanism. As such, acetazolamide-induced NCPE must remain a diagnostic consideration even when clinical suspicion is heavily biased toward hydrostatic causes, as failure to recognize this drug-induced etiology may delay essential therapeutic interventions. Furthermore, to the best of our knowledge, this article represents the first comprehensive synthesis of all previously reported cases of acetazolamide-associated acute respiratory failure. By integrating these disparate clinical observations with their proposed pathophysiological mechanisms, which range from immune-mediated endothelial injury to capillary leak syndromes, this review provides a unified framework to facilitate earlier recognition and optimize perioperative safety in ophthalmic practice.

## Figures and Tables

**Table 1:** Laboratory values obtained at the time of admission.

Parameter	Result	Reference Range	Unit
White blood cell count	9.04	4.0–11.0	×10^9^/L
Neutrophil count	7	1.7–7.5	×10^9^/L
Lymphocyte count	1.26	1.0–4.5	×10^9^/L
Monocyte count	0.81	0.2–0.8	×10^9^/L
Eosinophil count	0.0	0.0–0.4	×10^9^/L
Basophil count	0.0	0.0–0.1	×10^9^/L
Haemoglobin	11	14–18	g/dL
Red blood cell count	3.8	4.20–6.30	×10^12^/L
Haematocrit	34.4	40.0–52.0	%
Mean cell volume	91.5	80–100	fL
Mean cell haemoglobin	28.6	27.0–33.0	Pg
Red cell distribution width	14.7	11.5–14.5	%
Platelet count	164	150–450	×10^9^/L
Prothrombin time	15.9	10.0–14.0	Sec
Activated partial thromboplastin time	47.7	22.0–35.0	Sec
Clauss fibrinogen level	4.5	2.0–4.0	μg/mL
D-dimer	0.3	0–0.5	μg/mL
High sensitivity troponin T	11	<58	pg/mL
BNP	96	H < 100	pg/mL
Total bilirubin	0.38	<1.20	mg/dL
Total protein	4.97	6.40–8.30	g/dL
Albumin	2.55	3.50–5.20	g/dL
Globulin	2.42	1.90–3.70	g/dL
Alkaline phosphatase	91	40–130	U/L
Alanine transaminase	11	<41	U/L
C-reactive protein	3.3	<0.5	mg/dL
Procalcitonin	0.47	<0.5	ng/mL
Presepsin	303	<314	pg/mL
Urea	97	17–49	mg/dL
Creatinine	1.5	0.7–1.2	mg/dL
Estimated GFR	47	>90	mL/min/1.73 m^2^
Sodium	145	133–146	mmol/L
Potassium	4.38	3.5–5.3	mmol/L

BNP: Brain Natriuretic Peptide; GFR: Glomerular Filtration Rate

**Table 2:** Acetazolamide-Associated Acute Respiratory Failure Cases (1992–2022).

Ref.	Date	Sex	Age	Acetazolamide Indication	Comorbidities	Acetazolamide Dosage	Onset to Symptoms	ICU Admission	Possible Underlying Mechanism	Outcome
[4]	2025	M	61	Vitrectomy	Unknown	250 mg PO	25 min	Yes	NCPE; increased capillary permeability	Discharged
[5]	2023	F	59	Cataract surgery	Anemia, Hypertension, Dyslipidemia, Diabetes mellitus type 2, COVID-19 infection	250 mg PO	30 min	Yes	NCPE; increased capillary permeability	Discharged
[7]	2017	M	61	Metabolic Acidosis	Cardiomyopathy, Chest wall instability	500 mg IV	1 h	Yes	NCPE; Drug-induced pulmonary capillary leak; acute inflammatory cascade	Discharged
[14]	2016	M	81	Cataract surgery	Chronic renal insufficiency	250 mg PO	45 min	Likely	Cardiogenic pulmonary edema	Discharged
[13]	2014	M	76	Cataract surgery	Hypertension	250 mg PO	30 min	Yes	NCPE; increased vascular permeability	Discharged
[6]	2013	F	80	Cataract surgery	Hypertension, Diabetes mellitus type 2, Obesity, Dyslipidemia, Hypothyroidism	250 mg PO	30 min	Yes	Possible hypersensitivity reaction	Discharged
[12]	2002	F	79	Cataract surgery	Hypertension	250 mg PO	20 min	Yes	Possible hypersensitivity reaction; NCPE could not be definitively excluded	Discharged
[11]	1998	M	70	Cataract surgery	Not provided	250 mg PO	30 min	Unknown	Presumed sulfonamide hypersensitivity reaction	Discharged
[10]	1992	F	66	Glaucoma	Not provided	Not provided	Not provided	Yes	Fulminant anaphylactoid reaction with laryngeal edema; airway compromise	Deceased

Abbreviations: M, Male; F, Female; PO, Per Os (oral administration); IV, Intravenous; ICU, Intensive Care Unit; NCPE, Non-Cardiogenic Pulmonary Edema; min, minutes; h, hour(s)

## Data Availability

The original data presented in the study are included in the article, further inquiries can be directed to the corresponding author.

## Abbreviations

The following abbreviations are used in this manuscript:

NCPE	Non-cardiogenic pulmonary edema
IOP	Intraocular pressure
ICU	Intensive Care Unit
SpO_2_	peripheral oxygen saturation
LV	Left ventricular
CT	Computed tomography
VAP	Ventilator-associated pneumonia
ARDS	Acute respiratory distress syndrome
PaO_2_	Partial pressure of oxygen
FiO_2_	Fraction of inspired oxygen
CTPA	Computed tomography pulmonary angiography
GFR	Glomerular filtration rate
IgE	Immunoglobulin E
DIPPD	Drug-induced pulmonary parenchymal disease
